# Fast-cycling unit of root turnover in perennial herbaceous plants in a cold temperate ecosystem

**DOI:** 10.1038/srep19698

**Published:** 2016-01-21

**Authors:** Kai Sun, M. Luke McCormack, Le Li, Zeqing Ma, Dali Guo

**Affiliations:** 1 Department of Ecology, College of Urban and Environmental Sciences, Laboratory for Earth Surface Processes of Ministry of Education, Peking University, Beijing 100871, China; 2 Center for Forest Ecosystem Studies and Qianyanzhou Station, Key Laboratory of Ecosystem Network Observation and Modeling, Institute of Geographic Sciences and Natural Resources Research, Chinese Academy of Sciences, Beijing 100101, China; 3 University of Chinese Academy of Sciences, Beijing 100049, China

## Abstract

Roots of perennial plants have both persistent portion and fast-cycling units represented by different levels of branching. In woody species, the distal nonwoody branch orders as a unit are born and die together relatively rapidly (within 1–2 years). However, whether the fast-cycling units also exist in perennial herbs is unknown. We monitored root demography of seven perennial herbs over two years in a cold temperate ecosystem and we classified the largest roots on the root collar or rhizome as basal roots, and associated finer laterals as secondary, tertiary and quaternary roots. Parallel to woody plants in which distal root orders form a fast-cycling module, basal root and its finer laterals also represent a fast-cycling module in herbaceous plants. Within this module, basal roots had a lifespan of 0.5–2 years and represented 62–87% of total root biomass, thus dominating annual root turnover (60%–81% of the total). Moreover, root traits including root length, tissue density, and biomass were useful predictors of root lifespan. We conclude that both herbaceous and woody plants have fast-cycling modular units and future studies identifying the fast-cycling module across plant species should allow better understanding of how root construction and turnover are linked to whole-plant strategies.

Fineroot production and turnover are key processes in global carbon and nutrient cycling, and may account for 20 to 33% of global terrestrial net primary productivity (NPP)^1^,^2^, yet the accuracy of root turnover estimates at both local and global scales has been intensely debated^3^,^4^ and remains unresloved^5^,^6^,^7^,^8^. Critical for resolving this debate is to clearly identify which roots are turning over at what rate^9^, or whether a group of roots have similar patterns of survivorship as an intergrated unit of turnover.

Recently, progress has been made in identifying the unit of fast turnover in woody root systems. Although traditionally defined fine roots (i.e., roots <2 mm in diameter) were thought to represent a single fast-cycling portion of root systems, it is now clear that fine roots in woody plants contain multiple branching levels with different morphological, chemical and demographic traits^6^,^10^,^11^,^12^. Accumulating evidence also supports that only the non-woody portion of the fine roots are replaced rapidly with a turnover rate of approximately 6 to 24 months^13^,^14^,^15^,^16^,^17^,^18^. In a few woody species, this non-woody portion of fine roots has been found to be an integrated fast-cycling unit, termed as “ephemeral root modules”^19^,^20^. So far, this module concept has only been applied to woody species where the separation between woody and non-woody roots corresponds to the dichotomy in tissue longevity and function. Whether there is a division separating different parts of root system in herbaceous plants are unclear because the entire root system in herbaceous species are thought to be mainly confined to primary anatomical structure and may lack secondary wood development^21^, thus we may not be able to use non-woody vs.woody dichotomy to seperate roots of different turnover rates in herbaceous species.

Herbaceous plants and woody plants differ markedly but also share similarities in patterns of above- and belowground tissue turnover^22^,^23^. Perennial herbaceous plants from cold climates often have cheaply-constructed aboveground tissue and the entire aboveground may be shed annually to avoid winter freezing^24^,^25^. By contrast, woody plants maintain their woody scaffolding (including stems and woody roots) year-round and only shed the ephemeral parts such as leaves or ephemeral roots. However, perennial herbaceous plants also maintain a persistent living tissue mainly at the juncture between the above and belowground, such as the rhizome or root collar which is required for the regeneration of stems and roots the next year. What we do not know is whether, in belowground, aside from rhizome or root collar, there is also a portion of root system (which is non-woody) in herbaceous plants that would persist through winter in cold environments. Resolving this question would provide insights on whether ephemeral root modules occur in both woody and herbaceous plants and the differences or similarities in the design of this root module.

In addition, direct observations of root dynamics and accurate estimates of root lifespan remain a challenging task. One solution is to identify key root traits as predictors for root lifespan in the absence of empirical survivorship data^26^,^27^. Root traits including root diameter, specific root length (SRL), tissue density, and N concentration have been found to be related with fine-root lifespan in both woody and herbaceous species^16^,^28^,^29^,^30^. However, whether these traits are applicable in predicting lifespan of different portions in the herbaceous root system have rarely been fully tested (but see ref.^28^).

In this study, we used *in situ* root windows to observe root production and mortality of seven dominant perennial herbaceous species in the understory of a 44-year-old larch (*Larix principis-rupprechtii*) plantation. These species occur widely in the understory of larch and natural birch forests and in wetter parts of grasslands throughout the region. They represent taxonomically diverse herbaceous species including one grass and six forbs, which varied widely in their finest root diameter (from 0.11 mm to 0.23 mm) and length (from 3 mm to 13 mm). Our first objective was to determine how belowground systems are designed in herbaceous plants whether there are fast-cycling units (ephemeral root modules) parallel to woody plants. We hypothesized that such ephemeral root modules do exist and each module is composed of multiple branching levels because herbaceous root systems lack secondary wood development and because herbaceous plants in cold environments may only maintain a small portion of belowground system during the non-growing season (e.g. winter) to reduce total respiratory cost for the entire plant to save the storage for the tissue regeneration the next spring. Our second objective was to determine which root traits are predictors of root lifespan and turnover across species. Based on the cost-benefit perspective applied to leaf lifespan^26^,^31^, we hypothesized that root lifespan is positively related to the investment of construction cost of individual roots or the fast-cycling root module^16^,^28^.

## Materials and Methods

### Site description

The study site is located at the Saihanba Research Station of Peking University, situated in Saihanba National Nature Reserve (42°24'03“N, 117°12'25“E, elevation 1505 m), Hebei Province, China. It is part of the typical forest-steppe ecotone in the temperate areas of East Asia. The mean January, July and annual temperatures are −21.8 °C, 16.2 °C, and −1.2 °C, respectively. The mean annual precipitation is 450 mm, with 398 mm occurring from April to September (unpublished data from Saihanba Research Station). The average frost-free duration over the past 40 years is 60.8 days. Winters are relatively long, with snow cover for approximately six months a year. The native vegetation of this area is paper birch and larch forest-steppe ecotone, which was heavily disturbed during the wartime in the 1940’s, leaving only bare land. This area was revegetated in the early 1960’s following a massive reforestation program started in 1962. The area is now dominated by forest plantations, and *Larix principis-rupprechtii* is the most widely planted and dominant species (38.7% of total forest area), covering an area of 38,670.84 ha (data from Saihanba Mechanised Tree Farm). Our field experiments were conducted in a *Larix principis-rupprechtii* plantation planted in 1966 with relatively flat topography. The soil is aeolian sandy material with carbon (C) and nitrogen (N) concentration of 18.2 ± 1.1 and 1.5 ± 0.01 (g/kg), respectively.

### Rhizotrons and image analysis

**Figure 1:**
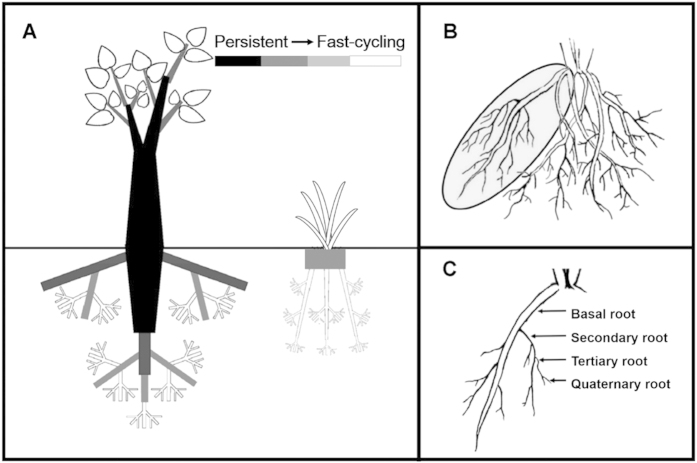
Schematic diagram of a typical woody plant with tap root system and a perennial herb plant with fibrous root system **(A)**, a fibrous root system of herb plant with a fast-cycling unit (an intact basal root and the fine lateral roots) outlined **(B)**, and a close-up of the fast-cycling unit indicating the developmental root classification used to dissect root segments of different branching levels (C).Black color indicates the permanent part of plant and grey color indicates a relatively low turnover rate of the organ (i.e. darker color indicates slower turnover). Organs in white are the fast-cycling units which turnover approximately every one to two years.

Our approach to dissect each root system was modeled after the developmental classifications of Barley (1970)^32^ (Fig. 1C), which allows for naming of basal roots (the largest roots on the rhizome or root collar) and subsequent lateral roots as they grew throughout the growing season. We identified the largest roots on the rhizome or root collar as basal roots, and its finer laterals as secondary and tertiary roots, and so on (Fig. 1C). With the developmental classification, basal roots could be classified consistently for all species and different species may have different levels of branching but always have basal roots. This developmental approach differs from the branching order approach which identifies the most distal root tip as the first order roots as work towards thicker roots^33^. To observe the root demography, we used the root window approach that was successfully used previously in the studies of root systems of both grasses^34^ and woody species^20^. We installed root windows under seven dominant deciduous perennial understory species including six forbs and one grass (Figure S1, Table 1). A total of 175 root windows were installed in May 2010, with 25 windows for each species. Each window was installed adjacent to a single individual of known species identity. For each of the randomly selected plants, the soil adjacent to one side of the plant was carefully swept away from the base of the plant to expose the stem and roots. After the basal roots were exposed, great care was taken to extract them and remove all lateral branches from the plant with minimal breakage. Then an ultra-clear glass window (10 cm by 10 cm) was carefully placed on the roots at an angle of 60° to 80° from horizontal in order to minimize changes in soil moisture. The depth observed by the windows covered the depth to which the majority roots were located for the species. To protect the roots and root windows during the observation intervals, a black plastic bag containing soil of equal weight to the soil removed to uncover the roots was placed on the window.

Patterns of root growth, development and mortality were monitored at each observation session throughout the growing season. Images of the rhizotrons were taken using a digital camera (G9; Canon, Tokyo, Japan) and used to monitor root longevity. Images were taken at intervals of 15 days from May to September in 2010 and four additional sets of images were collected in May and September in 2011, and in May and June in 2012. The lifespan of each individual root was defined as the elapsed time between its first appearance and death. A root was considered dead when it turned black and shriveled or disappeared^12^. The accuracy of these visual cues was confirmed by TTC (2, 3, 5-triphenyl tetrazolium chloride) vitality staining tests of roots, and ranged from 69% to 100% with a mean accuracy of 92% (Table S1). Prior to lifespan analysis, all roots observed in the images were classified into different branching levels (Fig. 1C). The branching level of each root was judged by the last image session before it died. Roots that were still present and alive at the last image session were counted as right-censored in survivorship analyses. The independent turnover/reoccurrence of a root was recognized if it was produced after half of all the roots from the same branching level died.

### Root sampling, dissection, morphology and chemistry

We measured key root traits related to the construction costs including individual root diameter, length, number, mass, specific root length, tissue density, and root nutrient (carbon and nitrogen) concentrations as well as the contributions of individual root branching levels to total root system length and biomass to investigate potential relationships among these functional traits and root lifespan. Among all root traits, individual root mass and number are considered most reflective of the overall construction costs on a mass basis. Root diameter and length together determine volume of individual roots which is generally positively related to root mass. Root tissue density reflects the construction costs on volume basis. Root nitrogen concentration is also considered a useful indicator root construction and maintenance costs.

Intact root systems of all seven species were harvested each month during the growing season in 2010 (May through August). Roots of 30–50 plants were sampled for each species each month to obtain sufficient mass for subsequent chemical analyses. Two extra sampling periods were conducted in August, 2010 and September, 2012, with 50 and 20 replicates for each species, respectively. These roots were used for morphological analyses (August, 2010) and TTC test procedures (September, 2012). Each plant was excavated within an intact soil block (50 × 50 × 40 cm^3^). A total of 1790 plants were sampled in this study.

**Figure 2:**
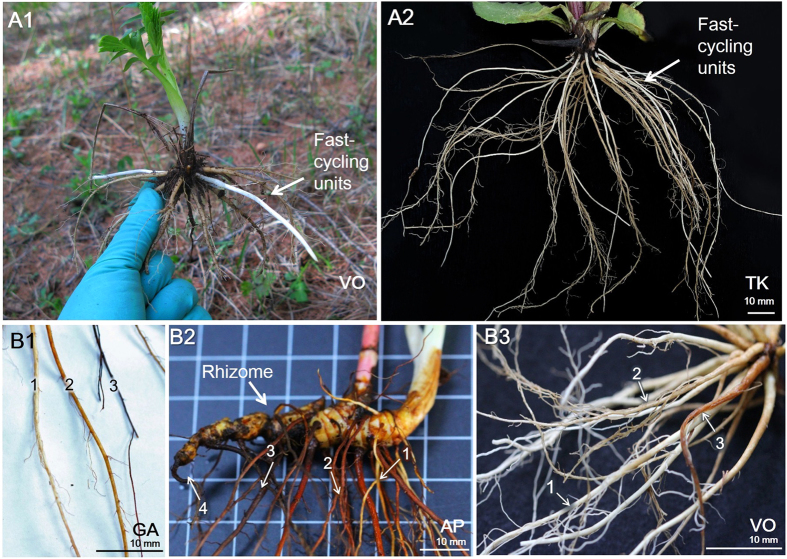
Images illustrating fast-cycling turnover units across seven herb species at different growth phases. Panel **(A)** shows typical root systems that were composed of repeated fast-cycling units with basal roots and their associated laterals in *Valeriana officinalis* (VO) and *Tephroseris kirilowii* (TK). Panel **(A)** also shows the emergence of new basal roots before laterals grew. Panel B shows that fast-cycling units were independent from each other, showing different growth phases with young/white (1), middle aged (2), senile (3) and dead (4) roots in *Geum aleppicum* (GA) (panel **B1**), *Agrimonia pilosa* (AP) (panel **B2**) and *V. officinalis* (VO) (panel **B3**). These images show that within each units, basal root and its laterals were at the same growth phases, whereas different units in a root system may vary in growth phases.

As the soil is aeolian and sandy textured, classified as entisols with loose crumb structure, most of the soil particles did not strongly adhere to root tissues and easily fell off from the roots (Fig. 2A). The remaining soil was then carefully rinsed from the surface of roots and then the harvested root system was placed in a mesh bag (200 micron mesh size) and rinsed with deionized water. Once sampled, the roots were immediately put in a cooler and transported to the laboratory within 4 h and frozen for dissection and morphological analysis at a later date.

Morphological analysis was conducted using the dissected roots from plants sampled in late August in 2010 for each species. First, the number of roots of each branching level for each plant was counted after dissection. Roots of each order were then scanned (Expression 10000XL, Epson, America) and analyzed with WinRhizo Pro 2004 software (Regent Instruments, Quebec, Canada) for the average root diameter and total root length. These roots were then placed in an oven and dried at 60 °C for 48 h and then weighed. Specific root length, root tissue density, individual root length and mass were calculated with total root mass, length, number, and average diameter.

We measured elemental concentrations using the roots from four samplings conducted during the 2010 growing season. The samples were put in the oven after dissection and dried at 60 °C for 48 h. Samples were ground and root C and N concentration were then measured using an elemental analyzer (Vario Microcube; Elementar, Hanau, Germany). The average values of C and N concentration for each branching level/species across the four sample dates were calculated and used for further data analyses. A subset of roots sampled in September 2012 were assayed by a modified TTC test procedure^35^,^36^,^37^ to test the reliability of the visual cues used in rhizotron image analysis. Root samples were transported to laboratory within 2 h immediately after being sampled. Roots were then divided into live and dead groups using visual cues as was done in the rhizotron windows and the number of alive (A_1_) and dead (D_1_) roots of each branching level and species was counted (Table S1). All roots were cut into 0.5 cm pieces, submerged in 10 ml of 0.6% (w/v) 2,3,5-triphenyltetrazolium chloride in 0.05 M Na_2_HPO_4_-KH_2_PO_4_ (pH 7.4) + 0.05% wetting agent (Triton X-100), and vacuum infiltrated for 5 min. Samples were then incubated at 30 °C for 24 h in dark. Finally, the root pieces were taken out from the tube and were cross–sectioned by hand. The color and the integrity of cellular structure were examined using the Motic optical microscopy system (SMZ 161, Motic, China). Roots with red color and good structural integration were considered to be alive whereas the others were considered dead. The number of alive (A_2_) and dead (D_2_) roots of each branching level and species was then counted. Accuracy of visual assessment was then calculated using following formula: Accuracy = (A_2_/A_1_)*100% or (D_2_/D_1_)*100% (Table S1).

### Data analysis

We tracked a total of 6311 roots through root windows for survivorship analysis. Roots which survived beyond the end of our observation period were censored in the subsequent analyses (right-censored). For those that existed before our first observation (i.e. basal roots), estimates of their birth dates were judged by their color and the overwintering features of roots. For example, for species whose basal roots consistently died at the end of the growing season (based on observations across multiple years including observations of emergence and color) it was assumed that basal roots that were present must have been produced early in spring as opposed to persisting from previous years. The remaining roots whose approximate birthdate could not be confidently estimated were classified as left-censored. The Kaplan–Meier model was used for survivorship analysis, from which the median lifespan was estimated^38^,^39^. The Tarone-Ware test was used to compare the survival curves of different categories of roots. Because of a low mortality rate (<50%), the median lifespan could not be estimated for basal roots of *Geum aleppicum* using Kaplan–Meier model. Instead, the observed mean value is given in Table S5 and used for further regression analyses.

Differences in root diameter, individual root length, individual root mass, total root length (per individual plant), total root mass (per individual plant), total root number (average number of roots per individual plant), SRL, tissue density and N: C ratio among different branching levels within same species were analyzed using Tukey test when homogeneity of variance assumptions were satisfied and Dunnett test when they did not meet required assumptions (SPSS 20.0 statistical software, USA). Variable coefficients of each branching level were calculated for root diameter, root length, tissue density, specific root length (SRL), individual root mass, total root mass, total root length, N concentration, C concentration and N: C ratio of all species.

Cox proportional hazard regression was used to calculate crude hazard ratios of root lifespan (i.e. the ‘crude’ ratio is the hazard ratio without other covariates included in the model) and identify root traits that had a significant effect on root lifespan. The root traits that had significant effect on root lifespan were then used to test the effects of covariates on root lifespan. In this analysis, each branching level of roots of each species was treated as an independent replicate for regression analysis and there were 14 replicates (basal and secondary roots) or 7 replicates (basal roots) in total as basal and secondary roots represented most of the annual root mass production. Then we selected the best combined model out of all possible models according to Akaike’s Information Criterion adjusted for small sample size (AICc) for predicting root lifespan based on root traits. Results were considered statistically significant at *P* < 0.05. Regression analyses were conducted in R 3.0.2 with *Base* and *MuMIn* Packages (R Core Team, 2013).

## Results

### Patterns of root architecture and replacement

The belowground systems are composed of root system and rhizome or root collar. The rhizome and root collar were not classified as part of the root system but rather they were considered as modified subterranean stem or as the common joint between roots and stem according to their traditional botanical descriptions. Root systems of the seven species were composed of repeating root clusters (units) sprouting from the swollen rhizome or root collar (Figs 1B, 2). Each root cluster was composed of a basal root and its associated laterals (Figs 1, 2). However, no smaller repeating clusters (units) with similar assemblies with each other was found growing on basal root or finer laterals (Fig. 2). Across these species, basal root and its laterals were almost at the same growth phases within each unit, whereas different units within a root system may vary greatly in growth phases (Fig. 2).

A basal root and its associated laterals constituted a fundamental unit of replacement (i.e. turnover) (Fig. 2). All the seven herbaceous species produced basal roots, and these basal roots further produced a single pulse of finer and shorter lateral roots (data not shown) to form a lateral root branching system with 3–4 levels of branching (Figs 1, 2). The finer laterals then usually died within the year, after this single main pulse of cohort, individual basal roots generally did not produce new cohort of daughter roots and generally died later in the season or in the following growing season (Table 1). The majority of the basal roots (77% on average, all species) were shed during the winter, leaving only rhizome or root collar and a small part of root system intact (Figs 1, 2). Although the parent basal roots usually lived longer than their daughter lateral roots, no reoccurrence of daughter roots of any branching level was observed during our study (Fig. 1, Table 1).

### Root life-history

We observed 518 basal roots, 520 secondary roots, 556 tertiary roots and 4717 quaternary roots in total through the rhizotron windows (for the definitions of different roots please see Fig. 1C). Root lifespan differed among species and branching levels. Across all species, median lifespan for basal roots ranged from 165d (*Thalictrum petaloideum*) to 771d (*Tephroseris kirilowii*), 60d (*T. petaloideum)* to 681d (*Rubus saxatilis*) for secondary roots, 60d (*T. petaloideum)* to 439d (*R. saxatilis*) for tertiary roots, and 45d (*T. petaloideum*) to 424d (*R. saxatilis*) for quaternary roots (Table 1).

### Morphology, chemistry, biomass and production

**Figure 3:**
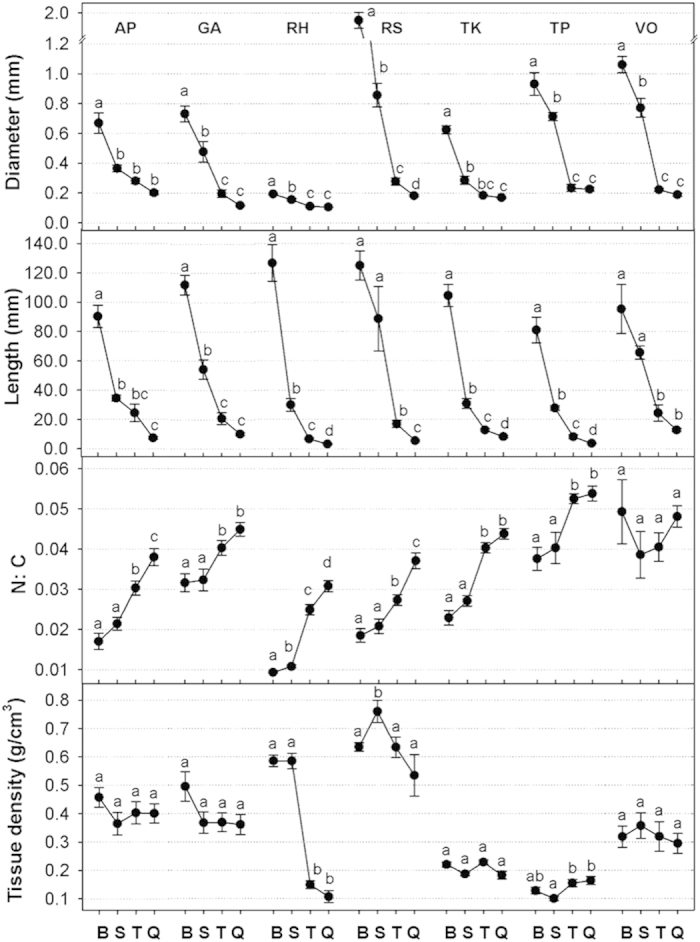
Root morphological traits for roots of different branching levels (basal root: B, secondary root: S, tertiary root: T, quaternary root: Q) from seven temperate perennial herb and grass understory species. Error bars represent one standard error of the mean. Lowercase letters within a species indicate significant (*P* < 0.05) differences among individual root branching levels. Abbreviations of species names are shown in Table 1.

**Figure 4:**
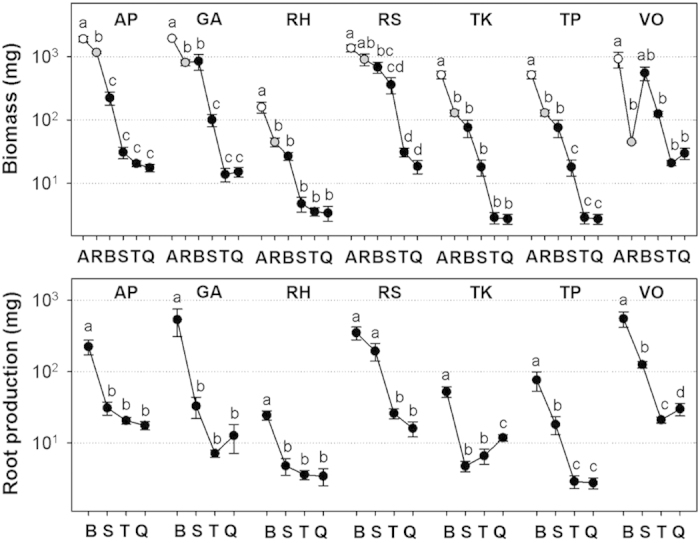
Total biomass of aboveground component (A, open circle), rhizome or root collar (R, grey circle), basal roots (B), secondary roots (S), tertiary roots (T) and quaternary roots (Q) and annual root production of each branching level among seven species. Lowercase letters within a species indicate significant (*P* < 0.05) differences among different plant components and each root branching level. Abbreviations of species names are shown in Table 1.

Root diameter for all root branching levels in all species was below 2 mm. Thus, the entire root systems for all seven species would fall within the traditionally-defined pool of fine roots (i.e. all roots less than 2 mm in diameter). However, root morphology and chemistry of individual roots varied significantly across different species or across different root branching levels in each species, similarly to woody species. Average root diameter and root length decreased from basal to quaternary roots (Fig. 3) whereas specific root length (SRL) increased significantly from basal to quaternary roots in all species. Root N concentration and N: C ratio also increased from basal to quaternary roots across all the species (Fig. 3). Root N concentrations of the basal and secondary roots exhibited roughly two-fold variation among all species (1.28–2.39%, 1.08–2.36%, respectively). Similar variation was found in N: C ratios. Patterns of tissue density differed among species, with some species displaying small variation across different root branching levels (e.g. *G. aleppicum, T. kirilowii, T. petaloideum,* and *Valeriana officinalis*), whereas other species expressed large variation across branching levels with general decreases in tissue density moving from basal to more distal branches (e.g. *Roegneria hondai,* and *R. saxatilis*). Total root length tended to be highest in quaternary roots. Total root number per individual plant increased whereas individual and total root mass decreased from basal to quaternary roots in all species (Fig. 4). Root numbers of basal roots were an order of magnitude lower than that of quaternary roots across all seven species. However, individual root mass of basal roots were two to four orders of magnitude higher than that of quaternary roots across all seven species, which results in one to two orders of magnitude more total root biomass of basal roots than that of quaternary roots in all species (Fig. 4). Overall, basal roots represented 62% to 87% of total standing root biomass (Table 1) and production of basal roots accounted for 60% to 81% of total annual root production across all species (Fig. 4). Annual root production of basal and secondary roots together accounted for 76% to 95% of total root production with an average of 88% across seven species (Fig. 4).

### Factors influencing root lifespan

Across all root branch levels and species, root lifespan increased with increasing root diameter, root length, tissue density and individual root mass, but decreased with increasing N: C ratio and total root number per plant (Table S2).

**Figure 5:**
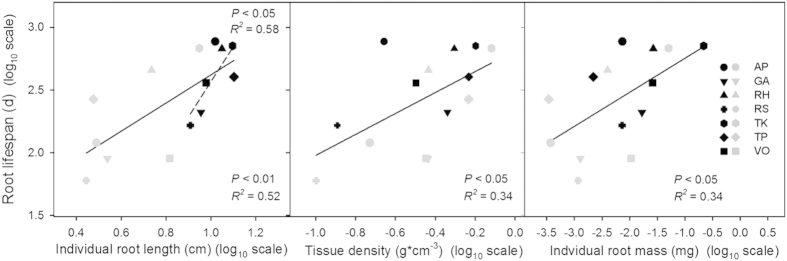
Regression plots of root traits (root tissue density, individual root length and mass) and root lifespan of basal and secondary roots on a log_10_ scale. Solid lines are the regression lines of root traits and root lifespan of basal and secondary roots (N = 14).The dashed line is the regression line of individual root length and root lifespan of basal roots only (N = 7). Symbols in black represent basal roots and symbols in gray represent secondary roots.

Patterns across basal and secondary roots were similar to those found across all root branching levels. Root lifespan increased with individual root length, tissue density, individual root mass, and total root number and decreased with N: C ratio across all species (Table 2, *P* < 0.0001). The best predictor models were selected from all possible regression models that included the above six variables based on ΔAICc and number of variables. The best model based on ΔAICc scores and *R*^*2*^ included root length alone (ΔAICc = 0, *R*^*2*^ = 0.52, *P* < 0.01). The second and third best models included tissue density (ΔAICc = 4.4, *R*^*2*^ = 0.34, *P* < 0.05) or individual root mass (ΔAICc = 4.5, *R*^*2*^ = 0.34, *P* < 0.05), respectively (Fig. 5).

For basal roots alone, root lifespan increased with root length, tissue density, individual root mass, and total root number and decreased with N: C ratio across all species. The best model based on ΔAICc scores and *R*^*2*^ included root length alone (ΔAICc = 0, *R*^*2*^ = 0.58, *P* < 0.05) (Fig. 5). Models incorporating more variables did not capture significantly more variation.

## Discussion

Belowground systems of perennial plants are complex and composed of multiple levels of branching with both the persistent tissue and the fast-cycling units. In woody species, woody framework roots are long-lived and only the distal two to three root order die within a few months to two years as fast-cycling units (or ephemeral root modules)^20^. However, in perennial herbs, whether there are fast-cycling units parallel to woody species remains unknown. Here, we found that in seven perennial herbs, rhizome and root collar are the persistent belowground tissue whereas the basal roots and its finer lateral roots form fast-cycling units, or the ephemeral root modules.

The ephemeral root module in perennial herbs studied here typically contain three to four orders of branching (Fig. 1), including a basal root, which is the largest root coming out of rhizome or root collar, and then secondary roots, or the finer laterals coming out of the basal root, and so forth. Because each basal root was the mother root for the finer laterals attached to it, the basal roots had to be born earlier and the finer lateral roots attached to them later. As such, there are some differences in root lifespan between basal roots and its finer laterals measured in root windows (Figure S2, Table 1, S5). However, each basal root produced its finer daughter roots only once in its life history and after this root production event it never produced other cohorts of daughter roots (Fig. 2, Table 1). Thus, there is no repeated turnover of daughter roots on the same basal root. We thus conclude that, despite the differences in root lifespan between basal root and its daughter roots, each basal root and its daughter roots form an integrated unit of root births and deaths, or a fast-cycling root module, thus supporting our first hypothesis.

Moreover, within each ephemeral root module, the basal roots constituted the majority of annual root biomass production and turnover (60–81%, respectively, Fig. 4). This dominance of total root turnover by basal roots was due to two reasons. First, despite the differences in root lifespan between basal roots and other lateral roots, they were all produced only once in the growing season, thus most of them had the same turnover rate (once a year) (Table 1). Second, basal roots represented a dominant portion (62–87%) of total root system biomass. The dominance of basal roots in total root turnover reinforces the idea that basal roots (and its finer lateral roots) behave as modular structures in herbaceous root systems studied here.

We also found that root lifespan of the fast-cycling unit was significantly correlated with root traits reflecting construction costs, supporting our second hypothesis. Specifically, root lifespan was positively related to root tissue density and average mass of an individual root (Fig. 5), indicating the total amount of carbon a plant invests into its roots is positively related to root lifespan. This is consistent with the long-held view that there is a coordination between root construction costs and root persistence, which is based on the tradeoff between C investment in an organ and the payback time to claim this investment (thus the duration of resource acquisition)^28^,^40^,^41^,^42^,^43^.

Fast turnover of the majority of the belowground system of the perennial herbs studied here is related to the whole-plant strategies in cold environments. Perennial herbs have full set of aboveground and belowground systems during the growing season. Once growing season is ended, these plants only maintain the rhizome, root collar, and a small portion of root systems which may serve as nutrient and carbon storage functions during non-growing season (Table S3). Our study site is characterized by harsh winters, during which only a small fraction of roots persisted over the dormant season (<30%). In such an ecosystem, carbon fixation is likely to be constrained by short growing season (i.e. ~60 frost-free days) and low growing season temperature (average growing season temperature of 10.5 °C)^44^. Therefore, the costs (relative to plant ability to fix C) of tissue maintenance should be high in such a system, and it appears that only a small fraction of root system are maintained to minimize the total maintenance costs for the entire plant. Besides, the existence of such a fast-cycling unit may be associated with low construction costs in herbaceous root systems, and it has been shown that low tissue construction costs are related to tissue shedding when encountering unfavorable environmental conditions (e.g. freezing or drought)^24^.

Apart from the similarity of belowground composition, there were also differences between the fast-cycling unit in herbaceous root systems and the “ephemeral root module” found in woody species. In woody species, all roots from the ephemeral root module were born and died simultaneously and fine root turnover was dominated by the finest roots in woody species (62%, data from Xia *et al.* 2010)^20^. Such differences may be related to the different roles of fast-cycling root module in whole-plant function. This was because in woody species, structural roots are very long-lived to ensure the anchorage of the aboveground stem and the storage of nutrients and carbohydrates, whereas only the smallest non-woody roots have fast turnover rates and function as ephemeral, absorptive roots^2^,^20^,^45^. For perennial herbs, however, both basal root and its associated finer laterals lack secondary development (e.g. development of cork cambium) and basal roots contributed about 25% to total root length in a root system (data not shown). And all basal roots and their lateral roots behave as ephemeral, absorptive roots and most of these roots may be shed in a year or two, with only rhizome or root collar being persistent belowground tissue, parallel to woody framework roots in woody plants.

The fast-cycling units (or ephemeral root modules) may be common in perennial herbaceous species from biomes with periodic unfavorable environmental conditions (Table S3). On the Alaskan tundra, Shaver and Billings (1975) reported that in the perennial herb *Eriophorum angustifolium*, the entire root systems were shed before the next growing season^46^. In a savanna-type ecosystem, West *et al.* (2003) found that *Schizachyrium scoparium* shed more than 80% of its roots before winter^47^. In a desert ecosystem, Nobel *et al.* 1992 showed “rain roots” in herbaceous species, which expressed similar characteristics to what we found in our study^48^. In temperate agroforestry systems, Van der Krift and Berendse (2002) showed that all roots of *Lolium perenne* L. died before winter following one year of observation^49^, whereas Watson *et al.* (2000) found that *Trifolium repens* shed 92–96% of its roots by the end of first growing season with only the stolon remaining alive^50^. In a semiarid ecosystem in Rush Valley, Peek *et al.* (2005) reported that most roots (>60%) of *Agropyron desertorum* died within the first year and all other roots died within the second year before winter^51^. We recognize that there are also other modes of root growth and replacement in perennial herbaceous species, particularly in those with tap root systems. For example, leguminous (e.g. *Medicago*) and umbelliferous plants (e.g. *Bupleurum*) often have tap roots that persist throughout their lifecycle, whereas *Aristida stricta* can tolerate harsh conditions rather than avoid them by shedding cheaply constructed tissues^46^. More studies testing fast-cycling units of root turnover among different types of herbaceous species are needed to provide a full understanding of root production and turnover across different biomes and different plant functional types.

Estimating root production and turnover of herbaceous plants has long been a challenge^5^,^52^,^53^,^54^. Our findings point to certain methodological modification for more accurately estimating root production and turnover for herbaceous root systems. First, based on our finding that 77% of the roots were born early in the growing season and then shed before the winter, we suggest that the ingrowth core method have the potential to accurately study root production in many herbaceous species within cold or dry seasonal environments. The ingrowth core method has been widely used for estimating root production and turnover^55^,^56^. However, ingrowth cores cannot be installed without imparting substantial artifacts associated with wounding existing active root systems which may be problematic in some systems^57^. For perennial herb species whose roots turn over rapidly, if the timing of ingrowth core installation precedes the pulse of growth in early spring, relatively accurate estimates of root production may be obtained. Still, we caution that successful use of the ingrowth core method will be contingent upon proper implementation. For example, it will be critical to install cores at the appropriate time. If this method was established in our study system only a few weeks following the onset of the growing season, the entire cycle of root production would have been missed for many of the species observed here.

Second, the maximum–minimum method can be another low-cost method to estimate root production for these herbaceous plants. This method uses differences in biomass between annual maximum and minimum fine root biomass to define annual root production^58^,^59^. The major uncertainty of this method is to ensure that data reflect appropriate periods of minimum and maximum root biomass and that there are no significant root deaths and births between consecutive sampling times. Based on our observations, 86% of all roots were produced before the middle of growing season and survived at least two months with very little mid-growing season root mortality (i.e. production and mortality were generally not coincident). Therefore, minimum levels of standing root biomass could be consistently captured by sampling before the onset of the spring growing season. The maximum production could then be appropriately captured after the middle of growing season without confounding effects of co-occurring mortality. It should be emphasized here that a full understanding of seasonal patterns of root growth and death are necessary for proper use of the maximum–minimum method for estimating root production and turnover.

## Conclusion

We demonstrated that each basal root and its attached finer laterals constituted a basic unit of root turnover among seven perennial herbaceous species. Specifically, basal roots dominated annual biomass production and turnover for the entire root system. Further studies are needed to examine whether such fast-turnover units occur in other perennial root systems, and to test the generality of the coordination between tissue construction costs and tissue persistence.

## Additional Information

**How to cite this article**: Sun, K. *et al.* Fast-cycling unit of root turnover in perennial herbaceous plants in a cold temperate ecosystem. *Sci. Rep.* **6**, 19698; doi: 10.1038/srep19698 (2016).

## Supplementary Material

Supplementary Information

## Figures and Tables

**Table 1:** Species information, survivorship, reoccurrence and contribution of each branching level to total root biomass and annual production in seven perennial understory species.

Species (abbreviation)	Life form	Family	Branch level	Median days	Lower 95%	Upper 95%	Reoccurrence (Y/N)	Percentage of total root mass (%)	Percentage of total root production (%)
*Agrimonia pilosa* (AP)	Forb	Rosaceae	B	210a	203	217	Y	76.4	76.4
			S	90b	84	96	N	10.6	10.6
			T	75b	71	79	N	7.0	7.0
			Q	60c	58	62	N	6.0	6.0
*Geum aleppicum* (GA)	Forb	Rosaceae	B	−a	–	–	Y	86.7	81.0
			S	454b	452	456	N	10.3	14.3
			T	439c	436	442	N	1.4	2.0
			Q	105d	102	108	N	1.6	2.7
*Roegneria hondai*(RH)	Grass	Gramineae	B	402a	179	625	Y	69.6	67.5
			S	267ab	27	507	N	12.4	13.2
			T	75b	70	80	N	9.3	9.9
			Q	75c	73	77	N	8.8	9.4
*Rubus saxatilis* (RS)	Forb	Rosaceae	B	711a	626	796	Y	62.3	59.7
			S	681b	352	1010	N	33.1	33.1
			T	439b	268	610	N	2.8	4.4
			Q	424c	372	476	N	1.7	2.7
*Tephroseris kirilowii* (TK)	Forb	Compositae	B	771a	700	842	Y	82.6	69.2
			S	120b	108	132	N	3.6	6.3
			T	120b	109	131	N	5.0	8.8
			Q	105b	100	110	N	8.9	15.7
*Thalictrum petaloideum*(TP)	Forb	Ranunculaceae	B	165a	160	170	Y	76.2	76.2
			S	60b	53	67	N	18.2	18.2
			T	60ab	54	66	N	2.9	2.9
			Q	45c	43	47	N	2.8	2.8
*Valeriana officinalis* (VO)	Forb	Valerianaceae	B	360a	356	364	Y	75.8	75.8
			S	90b	87	93	N	17.2	17.2
			T	90b	79	101	N	2.9	2.9
			Q	75c	73	77	N	4.1	4.1

Different lowercase letters following the number of median days indicate significant differences among branching levels (B: basal root, S: secondary root, T: tertiary root, Q: quaternary root) within the same species according to the Tarone-Ware test at *P* < 0.01. Upper and lower 95% refer to confidence intervals for median lifespan. Due to low mortality, the median life span could not be estimated for basal roots of *Geum aleppicum* using Kaplan–Meier model. Median lifespan relates to root survivorship. Reoccurrence of a root means that it was produced after half of all the roots from the same branching level died by its parent root. Percentage of total root mass/production refers to the percentage of total root mass/production of a given branch level to that of the whole root system.

**Table 2:** Summary of Cox proportional hazards for crude model fit for effects of root traits on root survivorship in basal and secondary roots.

Root traits	Basal and secondary roots			Basal roots		
	χ2	*P* > χ2	Risk ratio	χ2	*P* > χ2	Risk ratio
Root diameter	1.00E-04	0.99	1.00	3.24	0.07	1.33
Individual root length	91.08	<0.0001	0.90	99.78	<0.0001	0.62
Individual root mass	29.74	<0.0001	3.70E-04	9.67	<0.001	7.55E-03
Tissue density	57.37	<0.0001	0.13	33.01	<0.0001	0.11
SRL	2.67	0.1	1.00	1.69	0.19	1.00
N: C ratio	70.04	<0.0001	1.56E + 14	21.93	<0.0001	5.17E + 09
Total root number	13.52	<0.001	0.99	21.15	<0.0001	0.96

## References

[b1] Jackson R. B., Mooney H. A. & Schulze E. D. A global budget for fine root biomass, surface area, and nutrient contents. Proceedings of the National Academy of Sciences of the United States of America 94, 7362–7366, 10.1073/pnas.94.14.7362 (1997). 11038557 PMC23826

[b2] McCormack M. L. *et al.* Redefining fine roots improves understanding of below-ground contributions to terrestrial biosphere processes. New Phytol. 207, 505–518, 10.1111/nph.13363 (2015). 25756288

[b3] Matamala R., Gonzalez-Meler M. A., Jastrow J. D., Norby R. J. & Schlesinger W. H. Impacts of fine root turnover on forest NPP and soil C sequestration potential. Science 302, 1385–1387, 10.1126/science.1089543 (2003). 14631037

[b4] Strand A. E., Pritchard S. G., McCormack M. L., Davis M. A. & Oren R. Irreconcilable differences: fine-root life spans and soil carbon persistence. Science 319, 456–458, 10.1126/science.1151382 (2008). 18218895

[b5] Ahrens B., Hansson K., Solly E. F. & Schrumpf M. Reconcilable differences: a joint calibration of fine-root turnover times with radiocarbon and minirhizotrons. New Phytol. 204, 932–942, 10.1111/nph.12979 (2014). 25196967

[b6] Gaudinski J. B. *et al.* Measuring and modeling the spectrum of fine-root turnover times in three forests using isotopes, minirhizotrons, and the Radix model. Global Biogeochemical Cycles 24, GB3029, 10.1029/2009GB003649 (2010).

[b7] Keel S. G. *et al.* Allocation of carbon to fine root compounds and their residence times in a boreal forest depend on root size class and season. New Phytol. 194, 972–981, 10.1111/j.1469-8137.2012.04120.x (2012). 22452424

[b8] Warren J. M. *et al.* Root structural and functional dynamics in terrestrial biosphere models – evaluation and recommendations. New Phytol. 205, 59–78, 10.1111/nph.13034 (2015). 25263989

[b9] Pregitzer K. S. Fine roots of trees - a new perspective. New Phytol. 154, 267–270, 10.1046/j.1469-8137.2002.00413_1.x (2002). 33873419

[b10] Guo D. L. *et al.* Fine root heterogeneity by branch order: exploring the discrepancy in root turnover estimates between minirhizotron and carbon isotopic methods. New Phytol. 177, 443–456, 10.1111/j.1469-8137.2007.02242.x (2008). 17944827

[b11] Pregitzer K. S. *et al.* Fine root architecture of nine North American trees. Ecol. Monogr. 72, 293–309, 10.2307/3100029 (2002).

[b12] Wells C. E. & Eissenstat D. M. Marked differences in survivorship among apple roots of different diameters. Ecology 82, 882–892, 10.2307/2680206 (2001).

[b13] Espeleta J. F., West J. B. & Donovan L. A. Tree species fine-root demography parallels habitat specialization across a sandhill soil resource gradient. Ecology 90, 1773–1787, 10.1890/08-0056.1 (2009). 19694127

[b14] Guo D. *et al.* Anatomical traits associated with absorption and mycorrhizal colonization are linked to root branch order in twenty-three Chinese temperate tree species. New Phytol. 180, 673–683, 10.1111/j.1469-8137.2008.02573.x (2008). 18657210

[b15] Guo D. L., Mitchell R. J., Withington J. M., Fan P.-P. & Hendricks J. J. Endogenous and exogenous controls of root life span, mortality and nitrogen flux in a longleaf pine forest: root branch order predominates. Journal of Ecology 96, 737–745, 10.1111/j.1365-2745.2008.01385.x (2008).

[b16] McCormack M. L., Adams T. S., Smithwick E. A. H. & Eissenstat D. M. Predicting fine root lifespan from plant functional traits in temperate trees. New Phytol. 195, 823–831, 10.1111/j.1469-8137.2012.04198.x (2012). 22686426

[b17] Riley W. J., Gaudinski J. B., Torn M. S., Joslin J. D. & Hanson P. J. Fine-root mortality rates in a temperate forest: estimates using radiocarbon data and numerical modeling. New Phytol. 184, 387–398, 10.1111/j.1469-8137.2009.02980.x (2009). 19694965

[b18] Valenzuela-Estrada L. R., Vera-Caraballo V., Ruth L. E. & Eissenstat D. M. Root anatomy, morphology, and longevity among root orders in Vaccinium corymbosum (Ericaceae). American Journal of Botany 95, 1506–1514, 10.3732/ajb.0800092 (2008). 21628158

[b19] Salguero‐Gómez R. & Casper B. B. Introducing short roots in a desert perennial: anatomy and spatiotemporal foraging responses to increased precipitation. New Phytol. 191, 173–183, 10.1111/j.1469-8137.2011.03679.x (2011). 21434929

[b20] Xia M., Guo D. & Pregitzer K. S. Ephemeral root modules in Fraxinus mandshurica. New Phytol. 188, 1065–1074, 10.1111/j.1469-8137.2010.03423.x (2010). 21058949

[b21] Esau K. Plant anatomy. Soil Science 75, 407 (1953).

[b22] Reich P. B. *et al.* Relationships of leaf dark respiration to leaf nitrogen, specific leaf area and leaf life-span: a test across biomes and functional groups. Oecologia 114, 471–482, 10.1007/s004420050471 (1998). 28307896

[b23] Schweingruber F. H. & Poschlod P. Growth rings in herbs and shrubs: life span, age determination and stem anatomy. Vol. 79 (Swiss Federal Research Institute WSL, 2005).

[b24] Zanne A. E. *et al.* Three keys to the radiation of angiosperms into freezing environments. Nature 506, 89–92, 10.1038/nature12872 (2014). 24362564

[b25] Freschet G. T., Aerts R. & Cornelissen J. H. C. A plant economics spectrum of litter decomposability. Functional Ecology 26, 56–65, 10.1111/j.1365-2435.2011.01913.x (2012).

[b26] Eissenstat D. M. & Yanai R. D. In Advances in Ecological Research, Vol 27 Vol. 27 Advances in Ecological Research (eds Begon M. & Fitter A. H.) 1–60 (1997).

[b27] Iversen C. M. Using root form to improve our understanding of root function. New Phytol. 203, 707–709, 10.1111/nph.12902 (2014). 25040729

[b28] Ryser P. The Importance of Tissue Density for Growth and Life Span of Leaves and Roots: A Comparison of Five Ecologically Contrasting Grasses. Functional Ecology 10, 717–723, 10.2307/2390506 (1996).

[b29] Tjoelker M. G., Craine J. M., Wedin D., Reich P. B. & Tilman D. Linking leaf and root trait syndromes among 39 grassland and savannah species. New Phytol. 167, 493–508, 10.1111/j.1469-8137.2005.01428.x (2005). 15998401

[b30] Withington J. M., Reich P. B., Oleksyn J. & Eissenstat D. M. Comparisons of structure and life span in roots and leaves among temperate trees. Ecol. Monogr. 76, 381–397, 10.1890/0012-9615(2006)076[0381:cosals]2.0.co;2 (2006).

[b31] Westoby M., Falster D. S., Moles A. T., Vesk P. A. & Wright I. J. Plant ecological strategies: some leading dimensions of variation between species. Annual review of ecology and systematics 33, 125–159, 10.1146/annurev.ecolsys.33.010802.150452 (2002).

[b32] Barley K. P. The configuration of the root system in relation to nutrient uptake. Adv. Agron 22, 159–201, 10.1016/S0065-2113(08)60268-0 (1970).

[b33] Fitter A. H. Morphometric analysis of root systems: application of the technique and influence of soil fertility on root system development in two herbaceous species. Plant, Cell & Environment 5, 313–322, 10.1111/1365-3040.ep11573079 (1982).

[b34] Bai W. M., Wang Z. W., Chen Q. S., Zhang W. H. & Li L. H. Spatial and temporal effects of nitrogen addition on root life span of Leymus chinensis in a typical steppe of Inner Mongolia. Functional Ecology 22, 583–591, 10.1111/j.1365-2435.2008.01403.x (2008).

[b35] Comas L. H., Eissenstat D. M. & Lakso A. N. Assessing root death and root system dynamics in a study of grape canopy pruning. New Phytol. 147, 171–178, 10.1046/j.1469-8137.2000.00679.x (2000).

[b36] Shirazi A. M. & Muir P. S. In vitro effect of formaldehyde on Douglas fir pollen. Plant, Cell & Environment 21, 341–346, 10.1046/j.1365-3040.1998.00275.x (1998).

[b37] Steponkus P. L. & Lanphear F. O. Refinement of the Triphenyl Tetrazolium Chloride Method of Determining Cold Injury. Plant Physiology 42, 1423–1426, 10.2307/4261172 (1967). 16656672 PMC1086741

[b38] Kaplan E. L. & Meier P. Nonparametric estimation from incomplete observations. Journal of the American Statistical Association 53, 457–481, 10.2307/2281868 (1958).

[b39] Majdi H. Changes in fine root production and longevity in relation to water and nutrient availability in a Norway spruce stand in northern Sweden. Tree Physiology 21, 1057–1061, 10.1093/treephys/21.14.1057 (2001). 11560819

[b40] Craine J. M., Froehle J., Tilman D. G., Wedin D. A. & Chapin I. F. S. The relationships among root and leaf traits of 76 grassland species and relative abundance along fertility and disturbance gradients. Oikos 93, 274–285, 10.1034/j.1600-0706.2001.930210.x (2001).

[b41] Craine J. M. & Lee W. G. Covariation in leaf and root traits for native and non-native grasses along an altitudinal gradient in New Zealand. Oecologia 134, 471–478, 10.1007/s00442-002-1155-6 (2003). 12647118

[b42] Ryser P. & Lambers H. Root and leaf attributes accounting for the performance of fast- and slow-growing grasses at different nutrient supply. Plant and Soil 170, 251–265, 10.1007/bf00010478 (1995).

[b43] Schläpfer B., Ryser P. & Schlapfer B. Leaf and Root Turnover of Three Ecologically Contrasting Grass Species in Relation to Their Performance along a Productivity Gradient. Oikos 75, 398, 10.2307/3545880 (1996).

[b44] Xia J. *et al.* Joint control of terrestrial gross primary productivity by plant phenology and physiology. Proceedings of the National Academy of Sciences 112, 2788–2793, 10.1073/pnas.1413090112 (2015). PMC4352779 25730847

[b45] Jia S., Wang Z., Li X., Zhang X. & McLaughlin N. B. Effect of nitrogen fertilizer, root branch order and temperature on respiration and tissue N concentration of fine roots in Larix gmelinii and Fraxinus mandshurica. Tree Physiology 31, 718–726, 10.1093/treephys/tpr057 (2011). 21849591

[b46] Shaver G. R. & Billings W. D. Root production and root turnover in a wet tundra ecosystem, Barrow, Alaska. Ecology 56, 401–409, 10.2307/1934970 (1975).

[b47] West J. B., Espeleta J. F. & Donovan L. A. Root longevity and phenology differences between two co-occurring savanna bunchgrasses with different leaf habits. Functional Ecology 17, 20–28, 10.1046/j.1365-2435.2003.00695.x (2003).

[b48] Nobel P. S., Alm D. M. & Cavelier J. Growth Respiration, Maintenance Respiration and Structural-Carbon Costs for Roots of Three Desert Succulents. Functional Ecology 6, 79–85, 10.2307/2389774 (1992).

[b49] Van der Krift T. & Berendse F. Root life spans of four grass species from habitats differing in nutrient availability. Functional Ecology 16, 198–203, 10.1046/j.1365-2435.2002.00611.x (2002).

[b50] Watson C. A. *et al.* Environment-induced Modifications to Root Longevity in Lolium perenne and Trifolium repens. Annals of Botany 85, 397–401, 10.1006/anbo.1999.1048 (2000).

[b51] Peek M. S., Leffler A. J., Ivans C. Y., Ryel R. J. & Caldwell M. M. Fine root distribution and persistence under field conditions of three co-occurring Great Basin species of different life form. New Phytol. 165, 171–180, 10.1111/j.1469-8137.2004.01186.x (2005). 15720631

[b52] Hendrick R. L. & Pregitzer K. S. The Demography of Fine Roots in a Northern Hardwood Forest. Ecology 73, 1094–1104, 10.2307/1940183 (1992).

[b53] Norby R. J. & Jackson R. B. Root dynamics and global change: seeking an ecosystem perspective. New Phytol. 147, 3–12, 10.1046/j.1469-8137.2000.00676.x (2000).

[b54] Trumbore S. E. & Gaudinski J. B. The secret lives of roots. Science 302, 1344–1345, 10.1126/science.1091841 (2003). 14631025

[b55] Flower-Ellis J. & Persson H. Investigation of structural properties and dynamics of Scots pine stands. Ecological Bulletins, 125–138 (1980). 10.1016/0378-1127(82)90041-X (1980).

[b56] Vogt K. A. & Persson H. In Techniques and approaches in forest tree ecophysiology (eds Lassoie J. P. & Hinckley T. M.) 477–501 (CRC Press, Inc., 1991).

[b57] Epron D., Farque L., Lucot E. & Badot P.-M. Soil CO2 efflux in a beech forest: the contribution of root respiration. Annals of Forest Science 56, 289–295, 10.1051/forest:19990403 (1999).

[b58] Nadelhoffer K. J. & Raich J. W. Fine Root Production Estimates and Belowground Carbon Allocation in Forest Ecosystems. Ecology 73, 1139–1147, 10.2307/1940664 (1992).

[b59] Vogt K. A., Vogt D. J. & Bloomfield J. In Root Demographics and Their Efficiencies in Sustainable Agriculture, Grasslands and Forest Ecosystems Vol. 82 Developments in Plant and Soil Sciences (ed Box JamesE Jr.) Ch. 61, 687–720 (Springer Netherlands, 1998).

