# Supplementary material for: Fast-cycling unit of root turnover in perennial herbaceous plants in a cold temperate ecosystem
Source: Sci Rep. 2016 Jan 21;6:19698. doi: 10.1038/srep19698 (PMC4726329; doi:10.1038/srep19698)

Fast-cycling unit of root turnover in perennial herbaceous plants in a cold temperate ecosystem

Kai Sun[1], M. Luke McCormack[2], Le Li[2, 3], Zeqing Ma[2], Dali Guo[2*]

Supplementary information

Table S1 Accuracy of visual assessment the status of roots (i.e. alive or dead) from different branching levels (P: basal, S: secondary, T: tertiary, Q: quaternary) in seven perennial understory species. Number of alive/dead roots by visual assessment means the number of alive/dead roots determined based on color and textual cues. Number of alive/dead roots by TTC staining is the number of alive/dead roots determined by 2, 3, 5-triphenyl tetrazolium chloride vital staining results.

Species	Root	Status	Number of alive/dead roots by visual assessment	Number of alive/dead roots by TTC staining	Accuracy
A. pilosa	B	Alive	64	55	0.86
		Dead	29	29	1.00
	S	Alive	51	44	0.86
		Dead	22	22	1.00
	T	Alive	44	44	1.00
		Dead	24	21	0.88
	Q	Alive	42	39	0.93
		Dead	25	23	0.92
G. aleppicum	B	Alive	36	31	0.86
		Dead	15	15	1.00
	S	Alive	33	26	0.79
		Dead	22	22	1.00

	T	Alive	39	38	0.97
		Dead	13	13	1.00
	Q	Alive	92	90	0.98
		Dead	50	49	0.98
R. hondai	B	Alive	50	42	0.84
		Dead	25	25	1.00
	S	Alive	40	31	0.78
		Dead	20	20	1.00
	T	Alive	40	29	0.73
		Dead	20	20	1.00
	Q	Alive	80	57	0.71
		Dead	40	40	1.00
R. saxatilis	B	Alive	50	41	0.82
		Dead	25	25	1.00
	S	Alive	40	37	0.93
		Dead	20	20	1.00
	T	Alive	40	38	0.95
		Dead	20	20	1.00
	Q	Alive	80	79	0.99
		Dead	40	36	0.90
T. kirilowii	B	Alive	50	45	0.90
		Dead	25	25	1.00
	S	Alive	40	37	0.93
		Dead	20	20	1.00
	T	Alive	40	39	0.98
		Dead	20	20	1.00
	Q	Alive	80	75	0.94
		Dead	40	40	1.00
T. petaloideum	B	Alive	54	39	0.72
		Dead	27	27	1.00
	S	Alive	42	29	0.69
		Dead	21	21	1.00
	T	Alive	52	44	0.85
		Dead	26	24	0.92
	Q	Alive	52	43	0.83
		Dead	26	21	0.81
V. officinalis	B	Alive	50	35	0.70

	Dead	25	25	1.00
S	Alive	40	33	0.83
	Dead	20	20	1.00
T	Alive	40	30	0.75
	Dead	20	20	1.00
Q	Alive	80	59	0.74
	Dead	40	40	1.00

Table S2 Regressions relating root morphology and chemical traits to lifespan.

Root trait	Slope	*P*	R^2
Root diameter	+	0.004	0.28
Root length	+	<0.001	0.48
N: C Ratio	-	0.019	0.19
Tissue density	+	<0.001	0.37
Log(SRL)	-	<0.001	0.36
Individual root mass	+	0.005	0.26
Log(Total root number)	-	0.008	0.24

Table S3 Characteristics of herbaceous species studied in other literature that may have similar fast-cycling units with species we studied.

Location	Ecosystem	*Species*	Growth habit	Root system type	Method	Turnover estimate	Reference
Alaska, USA	Tundra	*Eriophorum angustifolium*	Graminoid	Fibrous	Root box	Annual (<1 yr)	Shaver and Billings 1975
Craibstone Estate, UK	*Prunus avium* garden	*Lolium perenne*	Graminoid	Fibrous	Minirhizontron	0.12 yr	
Craibstone Estate, UK	*Prunus avium* garden	*Trifolium repens*	Graminoid	Fibrous	Minirhizontron	0.11 yr	Watson et al. 2000
Apennine Mountains, ITA	*Acer pseudoplatanus* garden	*Lolium perenne*	Graminoid	Fibrous	Minirhizontron	0.04 yr	
		Trifolium repens	Graminoid	Fibrous	Minirhizontron	0.03 yr	
Regina, CAN	Monoculture (common circumpolar species)	*Festuca rubra*	Graminoid	Fibrous	Minirhizontron	0.13 yr	Pärtel and Wilson 2001
Colorado, USA	Steppe	*Bouteloua gracilis*	Graminoid	Fibrous	Minirhizontron	0.9 yr	Gill et al. 2002
		Bouteloua gracilis	Graminoid	Fibrous	Minirhizontron	0.5 yr	
Wageningen, NL	Monoculture	*Lolium perenne*	Graminoid	Fibrous	Minirhizontron	0.3 yr	Van der Krift and Berendse 2002
		Arrhenatherum elatius	Graminoid	Fibrous	Minirhizontron	0.8 yr	
		Nardus stricta	Graminoid	Fibrous	Minirhizontron	1.0 yr	
		Molinia caerulea	Graminoid	Fibrous	Minirhizontron	1.1 yr	
Waarde, NL	Salt marsh	*Elymus pycnanthus*	Graminoid	Fibrous	In-growth cores	0.3 yr	Bouma et al. 2003
		Spartina anglica	Graminoid	Fibrous	In-growth cores	1.0 yr	
South Carolina, USA	Oak forest	*Aristida stricta*	Graminoid	Fibrous	Rhizotrons	2.1 yr	West et al. 2003
		Schizachyrium scoparium	Graminoid	Fibrous	Rhizotrons	1.0 yr	
Utah, USA	Sagebrush-dominated	*Agropyron desertorum*	Graminoid	Fibrous	Minirhizontron	0.15 yr	Peek et al. 2005

	arid ecosystem						
		Agropyron repens	Graminoid	Fibrous		0.14 yr	
	Oak savannah, prairie, hardwood forest, pine forests and abandoned agricultural fields	*Koeleria cristata*	Graminoid	Fibrous		0.26 yr	
		Poa pratensis	Graminoid	Fibrous		0.25 yr	
		Andropogon gerardii	Graminoid	Fibrous		0.2 yr	
Minnesota, USA		*Schizachyrium scoparium*	Graminoid	Fibrous	In-growth and in situ root sampling	0.2 yr	Tjoelker et al. 2005
		Sorghastrum nutans	Graminoid	Fibrous		0.16 yr	
	Oak savannah, prairie, hardwood forest, pine forests and abandoned agricultural fields	*Anemone cylindrica*	Forb	Fibrous		0.18 yr	
		Asclepias syriaca	Forb	Fibrous		0.12 yr	
		Rudbeckia serotina	Forb	Fibrous		0.13 yr	
California, USA	Desert	*Agave deserti*	Forb/subshrub	Fibrous	Root chamber	< 0.25 yr	Nobel et al. 1992

Table S4 Glossary

Basal roots:

Roots that directly attached to the base of stem (rhizome or stolon), or the most proximal roots among all levels of root branching.

Rhizome:

An underground stem with nodes, can produce both roots shoots.

Root collar:

Specialized swollen area near stem apex in terrestrial plants.

Table S5

Species (abbreviation)	Branch level	Mean time (d)	Number observed	Number censored
Agrimonia pilosa (AP)	B	393	101	35
	S	104	24	1
	T	82	175	0
	Q	69	1079	0
Geum aleppicum (GA)	B	675	95	72
	S	394	301	60
	T	377	87	12
	Q	236	1217	7
Roegneria hondai(RH)	B	359	56	20
	S	300	21	5
	T	198	87	14
	Q	142	804	78
Rubus saxatilis (RS)	B	693	23	13
	S	555	14	6
	T	448	24	9
	Q	346	164	13
Tephroseris kirilowii (TK)	B	624	96	66
	S	307	33	7
	T	318	113	26
	Q	265	572	62
Thalictrum petaloideum (TP)	B	205	48	0
	S	62	17	0
	T	53	24	0
	Q	44	303	0
Valeriana officinalis (VO)	B	353	99	0
	S	119	110	0
	T	154	46	0
	Q	93	578	0

Figure S1 Example of rhizotrons we installed *in situ*. Panel A: the root window was under a plant with known identity. Panel B: a close-up view of a root window with roots grew in.

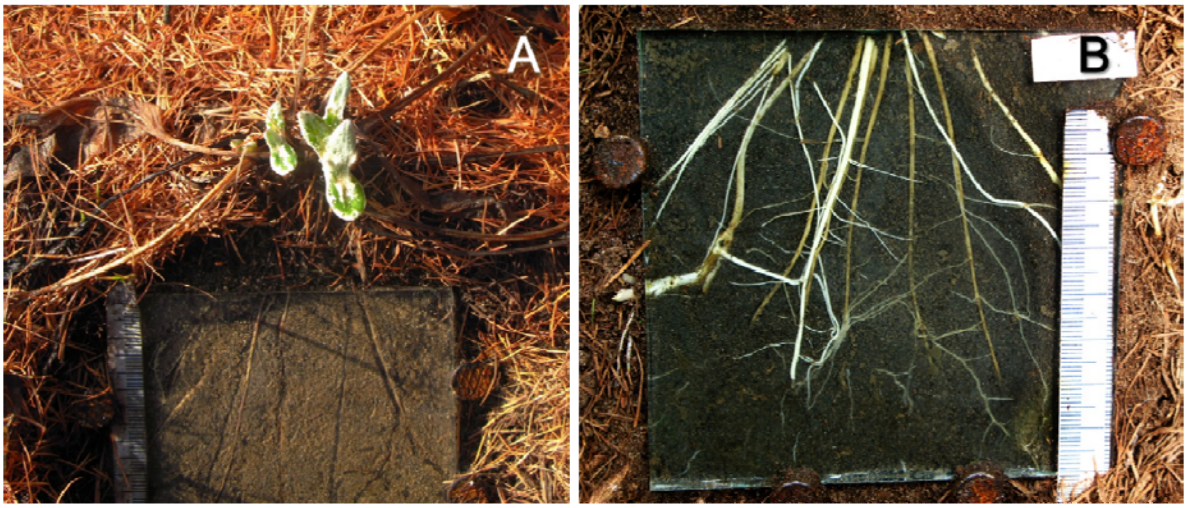

Figure S2 Root survivorship of seven temperate perennial understory species during the study period.

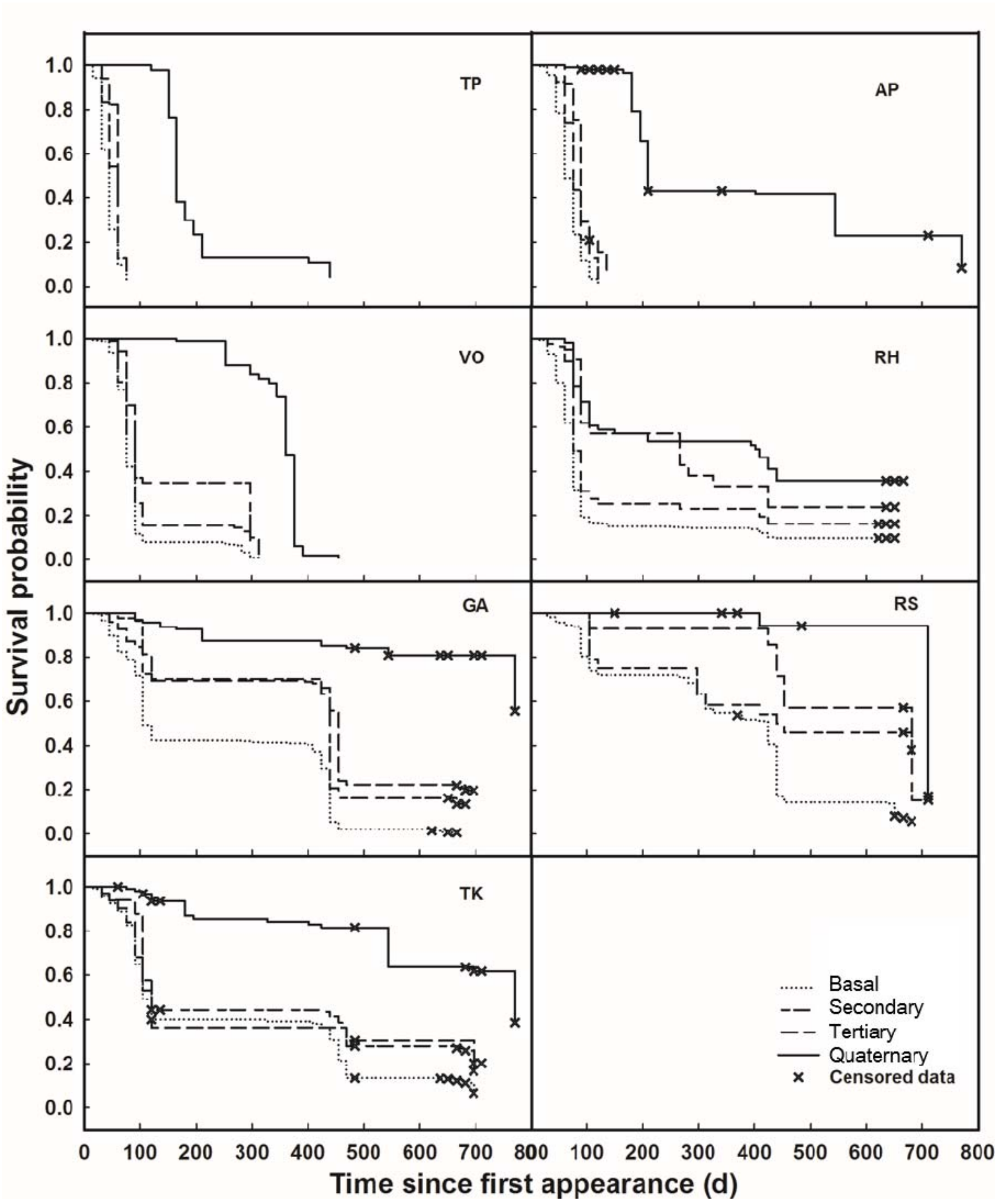

Supplement: Supplementary Information [file srep19698-s1.pdf]
